# Updating the mechanisms of common fragile site instability: how to reconcile the different views?

**DOI:** 10.1007/s00018-014-1720-2

**Published:** 2014-09-24

**Authors:** Benoît Le Tallec, Stéphane Koundrioukoff, Therese Wilhelm, Anne Letessier, Olivier Brison, Michelle Debatisse

**Affiliations:** 1 Institut Curie, Centre de Recherche, 26 rue d’Ulm, 75248 Paris Cedex 05, France; 2 UPMC University Paris 06, 75005 Paris, France; 3 CNRS UMR 3244, 75248 Paris, France

**Keywords:** Chromosome instability, Replication, Cell cycle, Cancer

## Abstract

Common fragile sites (CFSs) are large chromosomal regions long identified by conventional cytogenetics as sequences prone to breakage in cells subjected to replication stress. The interest in CFSs came from their key role in the formation of DNA damage, resulting in chromosomal rearrangements. The instability of CFSs was notably correlated with the appearance of genome instability in precancerous lesions and during tumor progression. Identification of the molecular mechanisms responsible for their instability therefore represents a major challenge. A number of data show that breaks result from mitotic entry before replication completion but the mechanisms responsible for such delayed replication of CFSs and relaxed checkpoint surveillance are still debated. In addition, clues to the molecular events leading to breakage just start to emerge. We present here the results of recent reports addressing these questions.

## Introduction

The replication process should be as reliable as possible in order to minimize mutations, but some regions of the genome, notably common fragile sites (CFSs), appear to raise specific problems. In recent years, their role in the generation of gross chromosome rearrangements has become increasingly evident, so that they are now recognized as major players in chromosome instability in cancer cells. Presently, there is a large consensus to consider that their fragility results from mitotic entry before completion of their replication. However, the mechanisms responsible for this delayed replication are still hotly debated. A common view is that CFSs are enriched in nucleotide sequences able to form secondary structures impeding fork progression. Stalled forks may then evolve into DNA breaks, the main source of chromosome rearrangements. However, new results have shown that the CFS setting is determined epigenetically, which strongly challenges this model. Not surprisingly, the frequency of breaks at CFSs is enhanced in cells deficient in ATR, the apical kinase that senses replication problems and triggers a signaling cascade that delays cell cycle progression, but how normal cells enter mitosis with incompletely replicated or damaged genome remains an important issue. We present here the results of recent works that shed some light on the epigenetic setting of CFSs and on the factors contributing to maintaining their stability.

## CFS instability is tissue dependent

CFSs are megabase-long chromosomal regions identified by conventional cytogenetics as loci prone to breakage in cells treated with low doses of aphidicolin, an inhibitor of replicative DNA polymerases (see D. Smith’s article in this issue). As previously suggested [1–4], recent mapping of CFSs in different cell types by conventional and molecular cytogenetic approaches confirmed that their setting is tissue dependent [5–7]. These results (i) imply that sequence features alone cannot account for CFS instability. (ii) raise the question of whether any chromosome region can be fragile in one or another type of tissue. The repertoire of CFSs is now available in lymphocytes, fibroblasts, breast and colon epithelial cells, and erythroid cells. Interestingly, comparison of these repertoires has revealed that approximately 50 loci account for all CFSs with break frequencies over 1 % found across these cell types. Strikingly, many of these loci are instable in several tissues, although their level of fragility could vary importantly from one cell type to the other [7]. Altogether these data suggest that a finite number of loci constitutes the pool of CFSs and that only a limited subset of these loci becomes fragile in a given cell type.

## Fragility correlates with the presence of large initiation-poor regions

Genome-wide analyses of replication timing [8] together with molecular combing have recently offered unprecedented opportunities to study the replication dynamics along CFSs. FRA3B, the most active CFS in human lymphocytes, was notably studied using both techniques [9]. In unperturbed lymphoblastoid cells, no differences were observed between FRA3B and the bulk genome in terms of fork speed and fork stalling. In aphidicolin-treated cells, fork speed was dramatically reduced, but again there were no differences between the bulk genome and the fragile region. Strikingly, mapping initiation events along FRA3B in unperturbed lymphoblastoid cells revealed an initiation-poor region extending over 700 kb, which coincides with the most fragile part of the site. Termination events were found all over this region, called the core, showing that replication completion is achieved upon merging of long-traveling forks emanating from each flanking region. In aphidicolin-treated cells, the core was again depleted of initiation events, but termination events were also infrequent, revealing a defect in replication completion. The latter observation can be explained by considering that fork speed reduction impacts long-traveling forks more profoundly than forks covering short distances, with particularly deleterious effects in late replicating regions such as FRA3B. Consistent results were obtained for FRA6E [10] and FRA16D [9]. By contrast, FRA16C displayed sequence-specific fork stalling and a high density of initiation whether the cells were treated with aphidicolin or not [11]. However, this site actually spans the same genomic region as FRA16B, which belongs to the category of rare fragile sites, the instability of which has long been shown to rely on micro- or mini-satellite repeats [12]. This makes it difficult to consider FRA16C a proper model for CFS instability.

## Similar epigenetic features set CFSs across cell types, but at different loci

The results described above for FRA3B, FRA16D and FRA6E raised intriguing perspectives because recent studies have shown that the choice of active replication origins evolves along with cell differentiation [13]. Remarkably, in fibroblasts, the density of initiation events was comparable in the core of FRA3B and in the bulk genome, and the site was shown to be quite stable in these cells [9]. In contrast, FRA1L and FRA3L, the two major CFSs in fibroblasts, display large origin-poor core regions in that cell type but not in lymphocytes, where they are stable [6]. These results strongly support the role of a paucity of initiation in CFS instability and show that commitment to fragility of major sites relies on the very same replication features in the different tissues, namely late replication and a paucity of replication initiation.

## Large genes constitute the pool of CFSs

It has long been reported that many CFSs co-map with very large genes, ranging from 600 kb to more than 2 Mb (reviewed in [14]; see also [15, 16]). The extensive CFS mapping performed recently in different human tissues and different species [7] together with improved annotation of human and mouse genomes have confirmed and extended this association, showing that between 80 and 100 % of human CFSs, depending on the cell type, and 100 % of those found in mouse embryonic fibroblast host genes over 300 kb long. Those genes are at least 15 times larger than the median length of human genes (~20 kb) and account for approximately 3 % of human genes. As an increasing number of previously non-annotated RNAs are being catalogued [17], it remains possible that the 15–20 % of CFSs devoid of large genes host yet to be identified large transcription units. Noticeably, CFSs mapped in chicken DT40 cells also correlate with large genes. The most fragile region in DT40 cells overlaps the large FAM190A and GRID2 genes and is therefore orthologous to human FRA4F and murine Fra6C1 [7]. These results suggest that the conservation of CFSs in vertebrates is linked to the conservation of large genes and conversely that chromosome regions containing large genes constitute the pool of potential CFSs for all cell types. The human genome contains approximately 700 such genes, sometimes organized in clusters. We thus calculated that the human pool of CFSs could be constituted of some 450 loci. Strikingly, re-analysis of the data provided by two reports that catalogued focal deletions in cancers and cancer cell lines [18, 19] has shown that large genes host 51.4 % of recurrent deletions and that many of these genes are associated with CFSs visible in one or the other tissues in which the sites have now been mapped [7]. These results lead to the conclusion that approximately half of the recurrent focal deletions found in human cancers originate from CFSs instable in the cell types from which the cancers derive.

## Transcription and fragile site setting

A recent study of five CFSs associated with large genes has suggested a correlation between instability and ongoing transcription, a hypothesis that accounts well for the tissue-dependence of fragility [20]. The authors have shown that transcription of genes extending over 800 kb takes more than one cell cycle, so that the transcription and replication machineries necessarily travel concomitantly on the same template. It was thus proposed that instability occurs when replication forks collide with R-loops, the structures formed by the association of the nascent transcript with the template DNA strand. Indeed, several lines of evidence now show that defects in mRNA processing increase genome instability in an R-loop-dependent manner, from yeast to mammalian cells (reviewed in [21]). However, comparison of RNA-seq data with the map of CFSs in HCT116 colon carcinoma cells has shown that the vast majority of large genes expressed in these cells, including those longer than 800 kb, are stable [7]. Thus, R-loop formation seems insufficient per se to set CFSs, although it may aggravate instability of otherwise committed regions. Nevertheless, the highly recurrent association of CFSs with large genes suggests a functional relationship that remains to be understand.

Another type of fragile regions was recently revealed in mouse splenic B cells upon treatment with hydroxyurea [22]. These sites, called ER-FSs (early replicating fragile sites), map to promoters of highly transcribed and early replicating genes. Importantly, orthologous regions of the human genome have been involved in approximately half of the amplifications and deletions recurrently found in biopsies of patients with diffuse large B cell lymphomas (see A. Nussensweig’s article in this issue). Altogether, the results link CFS and ER-FS instability to different types of replication stresses arising at least in part from conflicts between replication and transcription. Since a vast majority of chromosome rearrangements found in cancer cells result from the instability of either CFSs or ER-FSs, deciphering the molecular mechanisms responsible for these conflicts now represents a major issue in the field.

## CFSs and DNA secondary structures

The actual contribution of *cis*-acting DNA sequences to CFS instability is still strongly debated. Early analyses of a few cloned CFSs have revealed that they contain subregions enriched in highly flexible AT-rich sequences with the potential to form secondary structures. It was thus proposed that these sequences impede replication fork progression, which may lead to fork collapse, then DNA breaks and ultimately to chromosome rearrangements (reviewed in [23]). In support of this model, it has since been repeatedly reported that various types of secondary structures, including AT-rich sequences such as those found in CFSs, can perturb replication fork movement in vitro and in vivo (reviewed in [24]). However, recent genome-wide analyses of CFS sequences have provided contrasted results regarding the presence of flexible AT-rich regions within these sites. Indeed, some reports claim that CFSs are highly enriched in flexible AT-rich regions [25, 26], while others fail to identify specific accumulation of such sequences in the sites [4, 27]. The question of whether flexible AT-rich sequences, when present in a given CFS, constitute preferential regions of breakage in vivo has been extensively addressed, also leading to inconsistent conclusions. On one hand, several analyses have shown that DNA sequences within or adjacent to deletion breakpoints contain AT-rich flexible motifs [28, 29], suggesting that these regions are prone to breakage. On the other hand, deletions that remove AT-rich flexible sequences in FRA16D [29] or FRA3B [30, 31] fail to suppress breaks at the corresponding site. Finally, the recently described tissue specificity of CFSs strongly argues against an exclusive role of *cis*-acting DNA features in CFSs instability.

To reconcile this whole set of results, we propose the following scenario: upon replication stress, forks traveling along the initiation-poor core of CFSs may further slow down or stall if they encounter impediments such as DNA secondary structures. These structures may thus constitute preferential boundaries for under-replicated regions and, consequently, appear as hotspots of breakage at mitosis. In the absence of site-specific barriers, stress-induced fork slowing is sufficient per se to prevent completion of replication of the large core of CFSs, which leads to more fuzzy distribution of the borders of under-replicated regions, and hence of the breaks. By contrast, cells displaying a high density of replication initiation in the core can rescue stalled forks, which allows completion of replication in all conditions and prevents mitotic breakage. In this hypothesis, trans-acting factors that help the replication machinery to cope with fork barriers are also expected to participate in CFSs stability. Consistently, several works have recently shown that depletion of the Rev3 subunit of pol zeta, or of pol eta and possibly of pol kappa, three specialized DNA polymerases that facilitate DNA synthesis through non-canonical DNA structures [24], increases CFS instability [32, 33]. Furthermore, the absence of adequate trans-lesion polymerases favors accumulation of DNA lesions such as abasic sites, which could also compromise completion of replication of the CFS core.

## How can normal cells enter mitosis with under-replicated CFSs?

Faithful duplication of the genetic information before chromosome segregation is fundamental to the maintenance of genome integrity. To coordinate replication with mitosis, the cells have evolved a global signaling network that senses problems arising in S phase, stabilizes stalled forks, delays mitotic onset and stimulates DNA repair and/or apoptosis (reviewed in [34]). Breaks at CFSs are a major source of genome instability in pre-neoplastic lesions (see V. Gourgoulis's article in this issue), but how checkpoint-proficient cells escape surveillance and continue cycling with an incompletely replicated genome has remained unclear. A recent report [35] has shown that moderate fork speed reductions resembling those eliciting breakage at CFSs still allow cell cycle progression. Chromatin loading of sensors and mediators of the ATR pathway occurs in these conditions, but neither CHK1 nor p53 is activated. Accordingly, the authors found that the replisome disassembles upon moderate fork slowing in cells depleted of ATR, but not in cells depleted of CHK1. Partial activation of the pathway thus takes steps against fork collapse but tolerates S-phase progression and mitotic onset with incompletely replicated genome under moderate stress.

## Behavior of under-replicated regions at mitosis and beyond

The fact that the checkpoint fails to delay mitotic onset when only a few long-traveling forks remain active raises questions about the fate of persisting replication intermediates. Interestingly, the frequency of anaphase bridges, thought to represent unresolved replication or recombination intermediates, increases markedly upon moderate replication stress. It has been observed that the BLM helicase coats the bridges, while FANCD2-FANCI foci mark their tips. In addition, daughter cells display lesions sequestered in 53BP1 nuclear bodies in the following G1 phase. Not surprisingly, CFS sequences are enriched in those bodies (reviewed in [36]). Two recent works [37, 38] reported that the endonucleases MUS81/EME1 and ERCC1, which contribute to processing a wide variety of DNA structures such as stalled forks and Holliday junctions, are involved in the maintenance of CFSs during mitosis. SNM1B/APOLLO, a nuclease involved in the FANC pathway, also contributes to stabilizing CFSs [39]. Together, these results strongly suggest that segregation of incompletely replicated chromosomes can still be rescued through accurate processing of non-replicated DNA and that formation of 53BP1 bodies favors faithful repair and/or replication completion in the next cell cycle.

## DNA damage response (DDR) and CFS instability

A large number of DDR proteins are involved in the maintenance of CFS stability, notably ATR, CHK1, BRCA1, RAD51, Claspin, FANC proteins and BLM (reviewed in [40]). Strikingly, depletion of most of these proteins leads to fork slowing, raising the question of whether they control the stability of CFS via their functions in the DDR or via their indirect impact on fork velocity. A recent work focusing on the impact of ATR or CHK1 depletion on genome stability [35] has shown that the increased frequency of breaks at CFSs in the absence of CHK1 is completely accounted for by fork slowing, while ATR function is crucial to both sustaining global fork progression and avoiding disassembly and Mre11-dependent resection of long-traveling forks. In addition, some of the large genes nested in CFSs, such as *FHIT* in FRA3B, *SPIDR/KIAA0146* in FRA8I and *WWOX* in FRA16D, have been involved in DNA surveillance and/or repair pathways (see K. Huebner’s and R. Aqeilan’s articles in this issue). Their early inactivation in precancerous lesions might therefore further enhance genome instability.

## Conclusion

Instability of major CFSs has now been associated with late replication combined with a paucity of initiation events along a large DNA sequence called the core. The core has consequently to be replicated by long-traveling forks coming from flanking regions. All impediments slowing the progression of these forks increase the risk that cells will reach mitosis before complete replication of the core, leading to deleterious effects on CFS stability. Among others, DNA secondary structures and R-loops as well as mutations affecting proteins that contribute to erase either type of barrier participate to under-replication of the core and therefore enhance CFS instability.

The striking association of CFSs with large genes indicates a major role of these genes in fragility, but further work is needed to decipher the relationships linking fragility and transcription at the molecular level. One possibility could be that CFS setting relies on large-scale chromatin domains shaped by the association of transcription control elements such as insulators, promoters, terminators and enhancers of their cognate genes. These flexible domains would govern local replication timing and origin density in the different cell types. Alternatively, ongoing transcription machinery could clear or impair the recruitment of the pre-replication complex along the core of the genes.
